# Heterogeneity of tumour mutational burden in metastatic NSCLC demonstrated by endobronchial ultrasound sampling

**DOI:** 10.3389/fonc.2023.1150349

**Published:** 2023-03-13

**Authors:** Tracy L. Leong, Christian Aloe, Savreet Aujla, Hao Wang, Velimir Gayevskiy, Marie-Liesse Asselin-Labat, Lesley-Ann Gray, Daniel Steinfort, Steven Bozinovski

**Affiliations:** ^1^ Department of Respiratory Medicine, Austin Health, Heidelberg, VIC, Australia; ^2^ Personalised Oncology Division, The Walter and Eliza Hall Institute of Medical Research, Parkville, VIC, Australia; ^3^ Faculty of Medicine, University of Melbourne, Parkville, VIC, Australia; ^4^ Department of Medical Biology, The University of Melbourne, Parkville, VIC, Australia; ^5^ School of Health & Biomedical Sciences, RMIT University, Bundoora, VIC, Australia; ^6^ Australian Genome Research Facility Ltd., Melbourne, VIC, Australia; ^7^ Department of Respiratory Medicine, Royal Melbourne Hospital, Melbourne, VIC, Australia

**Keywords:** bronchoscopy, biomarker, lung cancer, tumour mutational burden, immunotherapy

## Abstract

**Introduction:**

Tumour mutational burden (TMB) is an important emerging biomarker for immune checkpoint inhibitors (ICI). The stability of TMB values across distinct EBUS tumour regions is not well defined in advanced lung cancer patients.

**Methods:**

This study included a whole-genome sequencing cohort (n=11, LxG cohort) and a targeted Oncomine TML panel cohort (n=10, SxD cohort), where paired primary and metastatic samples were obtained by endobronchial ultrasound transbronchial needle aspiration (EBUS-TBNA).

**Results:**

The LxG cohort displayed a strong correlation between the paired primary and metastatic sites, with a median TMB score of 7.70 ± 5.39 and 8.31 ± 5.88 respectively. Evaluation of the SxD cohort demonstrated greater inter-tumoural TMB heterogeneity, where Spearman correlation between the primary and metastatic sites fell short of significance. Whilst median TMB scores were not significantly different between the two sites, 3 out of 10 paired samples were discordant when using a TMB cut-off of 10 mutations per Mb. In addition, *PD-L1* copy number and *KRAS* mutations were assessed, demonstrating the feasibility of performing multiple molecular tests relevant to ICI treatment using a single EBUS sample. We also observed good consistency in *PD-L1* copy number and *KRAS* mutation, where cut-off estimates were consistent across the primary and metastatic sites.

**Conclusions:**

Assessment of TMB acquired by EBUS from multiple sites is highly feasible and has the potential to improve accuracy of TMB panels as a companion diagnostic test. We demonstrate similar TMB values across primary and metastatic sites, however 3 out of 10 samples displayed inter-tumoural heterogeneity that would alter clinical management.

## Introduction

Genomic heterogeneity within solid tumours contributes to disease relapse in lung cancer patients, where resistant clones become dominant under treatment pressures. The tumour mutational burden (TMB) represents a marker for genomic heterogeneity, which is defined as the number of somatic mutations per Mb of interrogated tumour exome sequence (1). Since tumour specific neoantigens arise from somatic mutations, tumours with a high TMB are likely to harbour more immunogenic neopeptides capable of eliciting a specific anti-tumour T cell response (2, 3). Smoking-related lung tumours notably harbour a high TMB, and a cut-off value of >10 mutations per Mb was predictive of improved response to immunotherapy in advanced NSCLC (4). TMB can be determined by whole-exome sequencing (WES), which detects nonsynonymous somatic mutations across the entire exome. Since the broad clinical application of WES to define TMB is challenging, NGS panels that screen for a smaller number of exonic mutations have been developed, which reduce costs and simplify the bioinformatic pipeline. The Harmonisation Project is developing strategies to achieve greater statistical calibration and reproducibility across TMB panel platforms (5).

In addition to technical variability, intra and inter-tumoural heterogeneity diminishes the accuracy and clinical applicability of companion immunotherapy diagnostic tests. Multi-site analysis of lung tumour biopsies reveals spatial heterogeneity in PD-L1 tumour staining within and across tumour sites, where sufficient variation could result in alteration of clinical management (6, 7). There is also heterogeneity relating to inter-observer variability in scoring PD-L1 staining, where harmonising testing procedures will be essential to improving reproducibility (8, 9). We have previously observed significant *PD-L1* mRNA expression heterogeneity across primary and metastatic sites, whereas *PD-L1* copy number proved more stable (10). In addition, segmentation of resection tumour samples revealed spatial heterogeneity of TMB scores that could alter clinical management, with greater heterogeneity observed when lymph node metastasis was included in the analysis (11). In this study, we investigated inter-tumoural TMB heterogeneity in advanced lung cancer patients using EBUS specimens, in contrast to most studies that use resection tissue from early-stage disease. We demonstrate significant heterogeneity across the primary and metastatic site to justify multi-site sampling for analysis of TMB levels.

## Methods

### Patient Cohorts

#### LxG cohort

The study was approved by the St Vincent’s Hospital human research ethics committee protocol number SVH14–256 and all subjects provided prospective written informed consent to participate in this study. EBUS-TBNA was performed on 11 patients, and patient characteristics are summarised in **Table 1**. **SxD panel cohort**. The biospecimens were acquired by Victorian Cancer Biobank, and the study was approved by the RMIT Human Ethics committee (SEHAPP 09-17). EBUS-TBNA was performed on 10 patients with advanced lung cancer as detailed in **Table 1**. Written informed consent was obtained from all participating individuals for the publication of any potentially identifiable images or data included in this article.

**Table 1:** Patient characteristics.

LxG Cohort						
ID	Age	Sex	Clinical stage	Smoking status	Pathology	Site sampled
1	78	F	IIIA	Ex	ADC	Primary LLL
						LN 7
2	50	M	IIIB	Ex	ADC	Primary RLL
						LN 7
3	70	M	IIIA	Ex	ADC	Primary RUL
						11R
4	69	F	IV	Ex	ADC	Primary R hilum
						LN 7
5	69	M	IV	Current	ADC	Primary RLL
						4R
6	68	F	IV	Never	ADC	Primary R hilum
						2R
7	64	M	IIIA	Ex	ADC	Primary RLL
						2R
8	68	M	IIIB	Ex	SQCC	LN 11L
						LN 2L
9	86	F	IIIA	Ex	SQCC	Primary LLL
						11L
10	55	M	IIIA	Current	SCLC	Primary RLL
						LN 7
11	48	F	IV	Ex	SCLC	Primary R hilum
						2R
SxD Panel Cohort						
ID	Age	Sex	Clinical stage		Pathology	Site sampled
1	85	M	IV	Ex	ADC	Primary RUL
						LN 8
2	60	M	IIB	Current	SQCC	Primary RLL
						LN 12R
3	69	M	IIIB	Ex	ADC	LN 11L
						LN 7
4	67	M	IV	Ex	ADC	Primary LLL
						LN 11L
5	68	F	IV	Ex	ADC	LN 4R
						LN 7
6	75	F	IIIA	Current	SQCC	Primary RLL
						LN 4R
7	71	F	IV	Current	ADC	Primary RLL
						LN 4R
8	63	F	IIIC	Never	ADC	Primary RML
						LN 2R
9	68	M	IV	Ex	ADC	Primary LLL
						LN 3P
10	71	M	IV	Ex	ADC	LN 11R
						LN 4R

### Determination of TMB

#### LxG panel cohort

EBUS-TBNA cytology samples containing > 20% tumour cells were processed for DNA extraction from the frozen cell suspension using the Qiagen (Hilden, Germany) DNeasy kit as per the manufacturer’s instructions. Sequencing was carried out on the Illumina (San Diego, CA, USA) HiSeq X Ten platform, as previously described (12). Variant calling files (VCFs) were filtered for poor-quality SNPs (depth < 15 and variant allele fraction (VAF) < 0.05) and known SNPs (dbSNP) to ensure scores were not biased by technical artefacts or germline variation. The ‘Ratio method’ was applied to define the ratio of callable mutations per Mb of callable exome. Three commonly employed exome definitions were applied; canonical exons (76.3 Mb), Agilent CREv2 exome (67.3 Mb) and TCGA exomes (38 Mb). The database herein referred to as ‘canonical’ consists of canonical exons downloaded from UCSC (https://genome.ucsc.edu/cgi-bin/hgTables). Regions were merged with bedtools to generate non-overlapping regions and filtered for gap regions in the assembly to capture WGS callable regions. Agilent ‘CREv2’ is the current industry standard for clinical whole exome sequencing. The ‘TCGA’ database includes regions from the deprecated Agilent All Exon V2 capture kit. The Canonical method was used to compare the primary and metastatic sites. Scores from each method were z-score standardised for comparison and inferential statistics and visualisations applied to aid interpretation.

#### SxD panel cohort

EBUS-TBNA cytology samples containing > 20% tumour cells were processed for DNA extraction from the frozen cell suspension using the AllPrep DNA/RNA/miRNA Universal kit (Qiagen, Germantown, MD). Briefly, frozen EBUS samples were suspended in RLT lysis buffer and DNA/RNA was extracted according to the manufacturer’s instructions. The NanoDrop One Microvolume UV-Vis Spectrophotometer (Thermo Fisher Scientific, Waltham, MA) was used to measure DNA/RNA yield and purity, as previously described (10). The Oncomine Tumor Mutation Load panel covering 1.7 Mb and 409 genes with known cancer associations was used. For library preparation, a total of 20 ng DNA input was amplified (15 cycles) and following quality control testing, libraries were sequenced on an Ion S5 GeneStudio XL/Prime (540 chip) for a single 200 bp run. The panels were analyzed using TorrentSuite and IonReporter, which determines mutational load by calculating the amount of tumour-specific somatic SNV mutations including splice-site mutations and intronic variants with a minimum coverage of 60x. Indels were excluded, and limit of detection was set to 5% minimum allele frequency. Germline filtering excluded polymorphisms by filtering against dbSNP, ExAC and 1000G using a cut-off of 0.0001 maximum population allele frequency. All variant calls were checked by manual inspection of the sequenced reads using the Integrative Genome Browser (IGV). *PD-L1* copy number was determined using the TaqMan Copy Number Assay kit (#Hs03704252), and *KRAS* mutation status was determined using the *KRAS* screening kit and the QX200 Droplet Digital PCR System (BioRad, Hercules, CA), as previously described (10). This kit includes seven KRAS G12/G13 mutations (G12A, G12C, G12D, G12R, G12S, G12V, G13D), and a cut-off of 0.2% mutant allele frequency was used to define presence of a KRAS mutation.

### Statistical analysis

All graphs were generated using GraphPad Prism version 9 (GraphPad Software Inc., San Diego, CA). Paired groups were evaluated using the nonparametric Wilcoxon test. Spearman correlation was performed to investigate associations between data. A p value of less than 0.05 was considered statistically significant.

## Results

**Figure 1:**
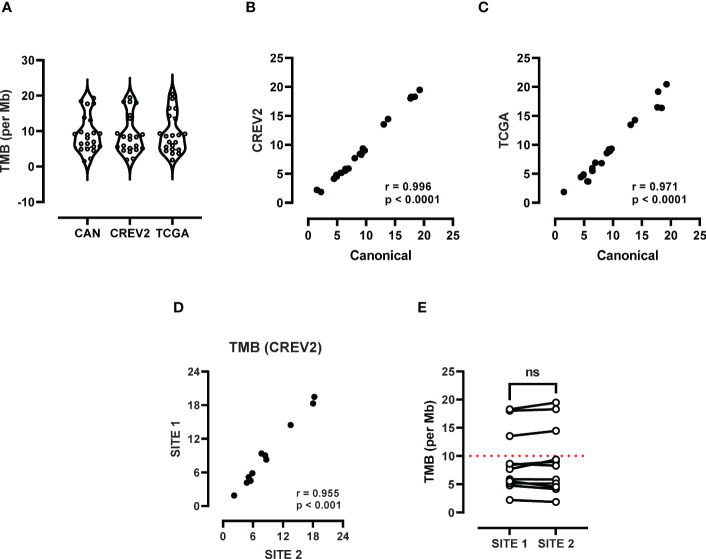
Assessment of TMB heterogeneity using whole exome sequencing. **(A)** TMB scores derived from three databases including TCGA, canonical and CREV2 were used to determine the number of exonic mutations per Mb. **(B)** Spearman correlation analysis was performed to investigate the association between TMB values derived from **(B)** TCGA versus the canonical database and the **(C)** TCGA and CREV2 databases. **(D)** TMB scores at the primary (site 1) and metastatic site (site 2) were compared by Spearman analysis and **(E)** paired analysis was performed between site 1 and site 2. ns, not significant.

TMB data was generated using a retrospective LxG cohort (**Table 1**), which included 5 female and 6 male patients with advanced disease (IIIA-IV), where 10/11 had a history of cigarette smoking. Cytology specimens were confirmed to contain at least 20% tumour cellularity, as previously described (12). TMB data generated through analysis of separate exome databases including Canonical, CREV2 and TCGA displayed a range of TMB values from 1.51 up to 20.44 mutations per Mb. Median TMB values of 8.50, 8.01 and 8.58 mutations per Mb for Canonical, CREV2 and TCGA respectively were not significantly different (**Figure 1A**). There was a strong positive correlation between the Canonical and the CREV2 databases (**Figure 1B**), as well as the TCGA database (**Figure 1C**). The Canonical database was subsequently used to compare TMB values across the two tumour regions. We investigated the association between TMB levels derived from the primary and metastatic sites by Spearman correlation, demonstrating excellent concordance (**Figure 1D**). Paired analysis of matching primary and metastatic specimens displayed consistent TMB values, where there was no significant difference in median TMB values across the sites (**Figure 1E**). When applying a TMB cut-off score of 10 mutations per Mb, the paired primary and metastatic samples were consistent in defining a low versus high score.

**Figure 2:**
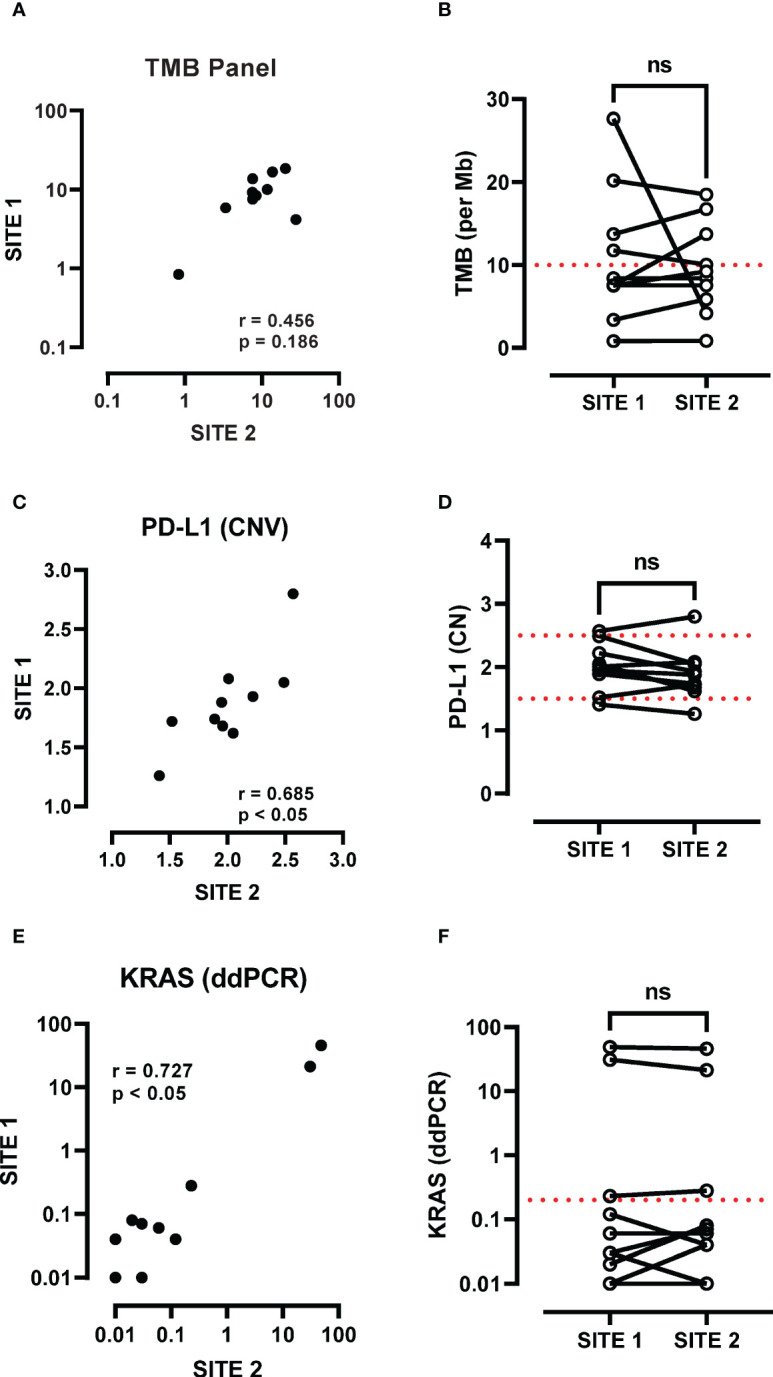
Assessment of TMB heterogeneity using the Oncomine panel. **(A)** TMB scores at the primary (site 1) and metastatic site (site 2) were compared by Spearman analysis and **(B)** paired analysis was performed between site 1 and site 2. **(C)** PD-L1 copy number at the primary (site 1) and metastatic site (site 2) were compared by Spearman analysis and **(D)** paired analysis was performed between site 1 and site 2. **(E)** KRAS G12/G13 mutations (G12A, G12C, G12D, G12R, G12S, G12V, G13D) were screened and a cut-off of 0.2% mutant allele frequency was used to determine presence of a KRAS mutation. KRAS mutation levels at the primary (site 1) and metastatic site (site 2) were compared by Spearman analysis and **(F)** paired analysis was performed between site 1 and site 2. ns, not significant.

The SxD panel cohort (**Table 1**) included 4 female and 6 male patients with advanced disease (IIB-IV), where 9/10 had a history of smoking. The correlation between TMB levels at the primary and metastatic sites fell short of significance when using the SxD panel specimens (**Figure 2A**). Median TMB values at the primary (8.0) and the metastatic (8.83) sites were not significantly different and were similar in value to the TMB score generated in the LxG cohort (7.70 and 8.31). In contrast to the LxG cohort, 3/10 samples were not concordant in defining whether a patient has a high TMB score based on a 10 mutation per Mb cut-off score (**Figure 2B**). Using the same tumour DNA samples, *PD-L1* copy number was measured, which was positively correlated across the primary and metastatic site (**Figure 2C**). There was no significant difference in *PD-L1* copy number between the paired primary and metastatic site (**Figure 2D**). We next determined *KRAS* mutation status by ddPCR. There was a strong positive association between the primary and metastatic site with respect to *KRAS* mutation levels (**Figure 2E**). *KRAS* genotyping detected 3/10 *KRAS* mutated patients based on a cut-off of 0.2% mutant allele frequency for this assay, and *KRAS* status was consistent across sites (**Figure 2F**).

## Discussion

Using EBUS-TBNA sampling and high-depth WGS, we have previously demonstrated genomic complexity in lung tumours that accumulate multiple mutations prior to the formation of frank malignancy (12). Whilst most known lung cancer driver genes were found to be highly conserved across sampling regions, there were multiple mutational events private to the primary or metastatic site (12). Here, we analysed whether these exceptions could influence the TMB score across primary and metastatic sampling sites. We utilised multiple filtering criteria and exome definitions to ensure robustness of our approach and observed that acquisition of private mutations did not influence the overall TMB calculation, which was highly concordant between the primary and metastatic sites. Our whole exome-based TMB findings are consistent with a model in which the majority of mutational events are acquired early and are maintained throughout the initiation and progression to metastasis.

We also demonstrate that TMB can be readily determined from small EBUS specimens when using a panel-based approach. Targeted panels are the preferred methodology for determining TMB due to lower costs and simplified bioinformatics pipelines. Our findings show that TMB levels are relatively stable across the primary and metastatic sites, however 3/10 samples displayed inter-tumoural heterogeneity that would alter clinical management based on a 10 mutation per Mb cut-off. The Oncomine TML panel screens for 409 nonsynonymous exonic mutations covering approximately 1.2 Mb, where this selective gene set may include mutations that evolve to become dominant during metastatic processes. Whether this is a consistent observation across alternative TMB panels with different gene sets requires further investigations. Variables such as panel reproducibility and tumour cellularity may also contribute to inter-tumoural heterogeneity when using EBUS derived samples. We propose that multi-site assessment of EBUS samples is highly feasible and should be considered to overcome variability when utilising TMB as a biomarker. In addition, we chose to freeze EBUS-TBNA samples following on-site evaluation because formalin-fixation has been shown to artificially increase TMB scores (13, 14). Further studies are needed to establish whether frozen EBUS-TBNA will outperform fixed EBUS-TBNA when using TMB as a biomarker for immunotherapy.

In summary, we demonstrate that it is highly feasible to utilise frozen EBUS-TBNA in patients with advanced lung cancer to quantify TMB and other putative immunotherapy biomarkers including *PD-L1* copy number and *KRAS* mutation status. It is highly plausible that a combination of markers may be needed to accurately predict immunotherapy responders and non-responders. Furthermore, a stepwise approach can be considered to minimise the number of tests including costly NGS panels. It is now evident that up to 50% of NSCLC patients positive for PD-L1 also harbour targetable driver mutations such as KRAS or EGFR (15). Since immunotherapy may be detrimental in the presence of EGFR mutations, TMB testing could be considered when the tumour is confirmed to be negative for EGFR and PD-L1. We propose that in addition to harmonising technical aspects relating to TMB panels, greater consideration relating to inter-tumoural variability across primary and metastatic sites is needed when developing a companion diagnostic for immunotherapy.

## Data availability statement

The datasets presented in this study can be found in online repositories. The names of the repository/repositories and accession number(s) can be found below: European Nucleotide Archive (https://www.ebi.ac.uk/ena), accession number PRJEB28616.

## Ethics statement

The studies involving human participants were reviewed and approved by RMIT Human Ethics committee (SEHAPP 09-17). St Vincent’s Hospital human research ethics committee protocol number (SVH14–256). The patients/participants provided their written informed consent to participate in this study. Written informed consent was obtained from the individual(s) for the publication of any potentially identifiable images or data included in this article.

## Author contributions

TL: Investigation, Methodology, Formal analysis, Writing - Original Draft. CA: Investigation, Methodology, Formal analysis, Writing – review & Editing. SA: Investigation, Formal analysis, Writing – review & Editing. HW: Investigation, Formal analysis, Writing – review & Editing. KR: Investigation, Writing – review & Editing. VG: Investigation, Formal analysis, Writing – review & Editing. M-LA-L: Formal analysis, Writing - Review & Editing, Supervision. LG: Methodology, Formal analysis, Writing - Review & Editing, Supervision. DS: Conceptualisation, Investigation, Formal analysis, Project administration, Writing - Original Draft. SB: Conceptualisation, Formal analysis, Writing - Original Draft, Supervision, Funding acquisition, Project administration. All authors contributed to the article and approved the submitted version.
